# No causal association between allergic rhinitis and migraine: a Mendelian randomization study

**DOI:** 10.1186/s40001-024-01682-1

**Published:** 2024-01-27

**Authors:** Hao Lv, Kunyu Liu, Yulie Xie, Yunfei Wang, Siyuan Chen, Peiqiang Liu, Mengting Guan, Jianchao Cong, Yu Xu

**Affiliations:** 1 Department of Otolaryngology-Head and Neck Surgery, Renmin Hospital of Wuhan University, 238 Jiefang Rd, Wuhan, 430060 Hubei China; 2 Department of Rhinology and Allergy, Renmin Hospital of Wuhan University, 238 Jiefang Rd, Wuhan, 430060 Hubei China; 3 Research Institute of Otolaryngology-Head and Neck Surgery, Renmin Hospital of Wuhan University, 238 Jiefang Rd, Wuhan, 430060 Hubei China; 4 Hubei Province Key Laboratory of Allergy and Immunology, Wuhan, 430071, Hubei China

**Keywords:** Mendelian randomization, Allergic rhinitis, Migraine, Causal relationship, Genetic correlation

## Abstract

**Purpose:**

Allergic rhinitis (AR) and migraine are among the most common public health problems worldwide. Observational studies on the correlation between AR and migraine have reported inconsistent results. This study aimed to investigate the causal relationship of AR with migraine and its subtypes, including migraine with aura (MA) and migraine without aura (MO).

**Methods:**

Bidirectional two-sample Mendelian randomization (MR) analysis was performed with publicly available summary-level statistics of large genome-wide association studies to estimate the possible causal effects. The inverse variance-weighted method was selected for primary analysis and was supplemented with the weighted median, weighted mode, and MR-Egger methods. The causal analysis using summary effect estimates (CAUSE) were further performed to verify the causality. Several sensitivity tests, including the leave-one-out, Cochran’s Q, MR-Egger intercept, and MR-PRESSO tests, were performed to assess the robustness of the results.

**Results:**

AR did not exhibit a significant causal correlation with the elevated risk of any migraine (odd ratio (OR), 0.816; 95% confidence interval (CI), 0.511–1.302; *P = *0.394), MA (OR, 0.690; 95% CI 0.298–1.593; *P = *0.384), or MO (OR, 1.022; 95% CI 0.490–2.131; *P = *0.954). Consistently, reverse MR analysis did not reveal causal effects of any migraine or its subtypes on AR. Almost all sensitivity analyses supported the robustness of the results.

**Conclusions:**

This MR study did not reveal a clear causal association between AR and migraine risk. More research is warranted to reveal the complex association between AR and migraine.

**Supplementary Information:**

The online version contains supplementary material available at 10.1186/s40001-024-01682-1.

## Introduction

Allergic rhinitis (AR), a common chronic inflammatory disease, is characterized by an enhanced response of the immune system to exogenous allergens. The pathophysiological mechanisms of AR are complicated and are related to an interplay between genetic predispositions and environmental factors [1]. The classic symptoms of AR include nasal itching, nasal congestion, watery rhinorrhea, and frequent sneezing, which have a negative impact on patients' health and quality of life [2]. The prevalence of AR has rapidly increased worldwide in recent decades. Globally, AR is estimated to affect 10–40% of adults and 5–15% of children [3].

Migraine is a highly prevalent neurovascular headache disorder associated with decreased productivity and marked disability [4]. According to the Global Burden of Disease Study 2016, the global prevalence of migraine is estimated to be 14% [5]. The typical clinical features of this neurological disorder include unilateral, throbbing headache attacks accompanied by nausea, vomiting, photophobia, and phonophobia [4]. However, migraine is a highly heterogeneous disorder and can be classified as migraine with aura (MA), migraine without aura (MO), and several rare subphenotypes based on the International Classification of Headache Disorders (ICHD) diagnostic criteria [6]. Although the pathogenesis of migraine has not been elucidated, it may be related to genetic factors and the activation of the trigeminovascular system [7].

Allergists and neurologists have been exploring the relationship between AR and migraine as these diseases exhibit high prevalence worldwide and overlapping symptoms [8]. Some pathophysiological features of AR are similar to those of migraine. For example, AR and migraine have a strong genetic component and similar triggers (e.g., inhaled irritants and weather changes) and inflammatory mechanisms [9]. However, the current clinical evidence for the association between AR and migraine has been inconsistent. Several population-based studies have demonstrated the correlation between AR and an increased risk and frequency of migraine [10–12], whereas other studies have suggested no correlation between AR and migraine [13, 14]. These previous observational studies could not demonstrate a causal relationship between AR and migraine owing to potential confounding factors and reverse causality [15]. The elucidation of the causal relationship between AR and migraine will enable the effective management of AR and migraine.

Mendelian randomization (MR) is a statistical approach that utilizes genetic variants, especially single-nucleotide polymorphisms (SNPs), to evaluate the potential causality between exposure and outcome [16]. The MR strategy is based on the principle of random assignment of alleles at meiosis. Thus, MR methods are independent of external factors confounding observational epidemiological studies [16]. Compared with traditional observational studies, MR analysis avoids confounders, reverse causality, and other biases. Furthermore, MR is recognized as an effective alternative to randomized controlled trials (RCTs) in determining causality in cases where economical, practical, and ethical RCTs are lacking [17]. Recently, the enhanced accessibility of human genetic data has increased the application of MR to infer causality in medical fields, such as etiological and drug target validation studies [18, 19]. This study conducted a bidirectional two-sample MR analysis to assess the potential causality between AR and migraine and its subtypes MA and MO.

## Materials and methods

### Study design

**Fig. 1:**
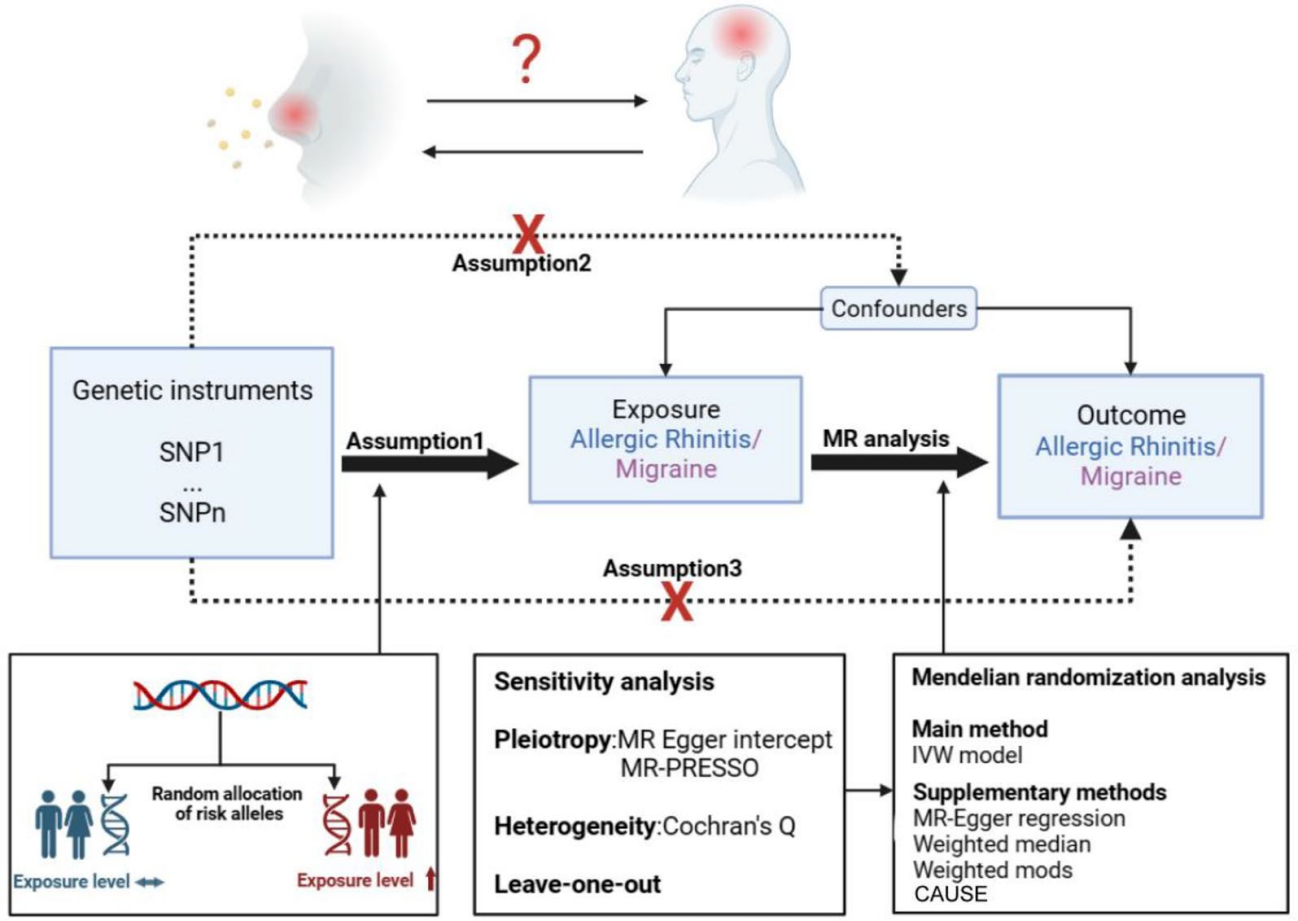
Schematic of MR study on the association between AR with migraine

SNPs were used as instrumental variables (IVs) to estimate the causal effect of exposure on outcome in this study. All SNPs included as IVs should fulfill the following three key assumptions for MR analysis [15] (Fig. 1): Assumption 1, IVs are strongly correlated with exposure; Assumption 2, IVs are not associated with any confounding factors related to both exposure and outcome; Assumption 3, IVs affect outcome only through exposure.

### Data sources

We obtained eligible summary-level statistics for each trait from large-scale, public GWAS datasets (Table 1). The GWAS summary data associated with AR, which were extracted from the UK biobank, comprised data of 25,486 patients diagnosed with AR and 87,097 controls. Summary-level statistics of migraine and its subtypes, including MA and MO, were extracted from the FinnGen consortium. The three summary-level statistics on migraine came from the same study, which involved 8,547 migraine cases, including 3,541 MA cases and 3,215 MO cases, and 176,107 controls. All participants were of European ancestry without overlap of exposure and outcome samples. No additional ethical approval was required as all data used in this MR study were obtained from previously published GWAS datasets.

**Table 1:** Summary of the data sources used in this MR study

Exposure or outcome	Sample size	Numbers of cases	Numbers of controls	Ancestry	Consortium	GWAS ID
AR	112,583	25,486	87,097	European	UK biobank	ukb-b-7178
Migraine	184,654	8547	176,107	European	FinnGen	finn-b-G6_MIGRAINE
MA	179,648	3541	176,107	European	FinnGen	finn-b-G6_MIGRAINE_WITH_AURA
MO	179,322	3215	176,107	European	FinnGen	finn-b-G6_MIGRAINE_NO_AURA

AR, allergic rhinitis; MA, migraine with aura; MO, migraine without aura; MR, Mendelian randomization analysis

### Selection of genetic variants as IVs

A series of quality control procedures was performed to identify genetic IVs that met these three MR assumptions [16]. The genome-wide significance threshold was set at P < 5 × 10^−8^ to screen for SNPs strongly associated with exposure. If SNPs did not satisfy the threshold, the *P*-value was relaxed to < 5 × 10^−6^ according to previous MR studies. Additionally, the clumping procedure (*R*^2^ < 0.001 and clumping distance > 10,000 kb) was performed to eliminate the effect of linkage disequilibrium between the included SNPs. Furthermore, traits related to SNPs were examined after the clumping process by querying the PhenoScanner database [20]. Common risk factors for migraines include smoking, alcohol consumption, systolic and diastolic blood pressure, body mass index, and major depression [21]. In addition, common risk factors for AR include bronchial asthma, nasal polyps, sinusitis, epistaxis, otitis media, and allergic strep throat according to previous MR studies [22]. SNPs associated with the confounders of outcome were excluded. Palindromic SNPs with intermediate allele frequencies were excluded by harmonizing exposure and outcome data to align SNPs on the same effect allele for both exposure and outcome. Moreover, the strength of all SNPs as genetic IVs was quantified using the F-statistic (F = β^2^ / se^2^). IVs with F-statistics less than 10 were considered weak IVs and were not used in subsequent MR analyses [23]. After the filtering procedure described above, these rigorously screened SNPs served as the final IVs for subsequent MR analysis.

### MR analysis

The causal association between AR and migraine was evaluated using four MR analysis methods. The inverse variance-weighted (IVW) method was the preferred method to determine the causality between exposure and outcome, while the MR-Egger, weighted median, and weighted mode methods served as alternative MR methods [16]. The IVW model can provide unbiased causal estimates when all IVs are valid [24]. The weighted median method offers an unbiased estimation in cases when up to 50% of the IVs are invalid [25]. When all IVs are invalid, the MR-Egger regression provides a conservative estimate of causality but with decreased statistical accuracy [26]. The weighted mode method can also be applied to evaluate the robustness of the MR results [27]. We also employed a Bayesian posterior probabilities-based MR method namely causal analysis using summary effect estimates (CAUSE) [28], as a further validation analysis of causality. The odds ratios (ORs) with corresponding 95% confidence interval (CI) were used to present the effect estimates from MR analyses. In addition, causal effects (i.e., OR) between AR and migraine were converted from a logit scale to a liability scale using the approach described by Byrne et al. [29], with an assumed population prevalence of 23% for AR [30] and 15% for migraine [31], respectively. The causal effect of exposure on the outcome was considered significant at *P* < 0.05.

Furthermore, a series of sensitivity analyses, including the pleiotropy test and heterogeneity test, was performed. The MR-Egger intercept test and MR-Pleiotropy Residual Sum and Outlier (PRESSO) global tests were used to detect potential horizontal pleiotropy [32, 33]. *P* > 0.05 indicated the lack of pleiotropy in IVs. The presence of horizontal pleiotropy can also be visualized with a funnel plot in which a symmetric graph suggests the lack of pleiotropy [34]. Heterogeneity between IVs was assessed using Cochran’s Q-test in the IVW and MR-Egger methods [35]. The effect of heterogeneity was disregarded if *P* > 0.05. Additionally, we performed the leave-one-out (LOO) analysis to check whether a single SNP was responsible for the causal association [36].

All these analyses were performed using R software (version 4.1.2) with the R packages TwoSample MR (version 0.5.7), MR-PRESSO (version 1.0), and CAUSE (version 1.2.0).

## Results

### Characteristics of the selected IVs

After screening, 31 significant SNPs related to AR (*P* < 5 × 10^−8^) that fulfilled the inclusion criteria were identified as valid IVs (Additional file 1: Table S1). As SNPs associated with migraine were not identified at the genome-wide significance threshold of *P* < 5 × 10^−8^, a less stringent threshold of *P* < 5 × 10^−6^ was used. Consequently, 12, 8, and 10 SNPs related to migraine, MA, and MO, respectively, were obtained as valid IVs (Additional file 1: Tables S2–S4). Of these, none of the SNPs were associated with relevant confounders. The F-statistics for all IVs were > 10, suggesting that the IVs included in this study were unlikely to be affected by weak instrument bias (Additional file 1: Tables S1–S4).

### Causal effects of AR on migraine

**Fig. 2:**
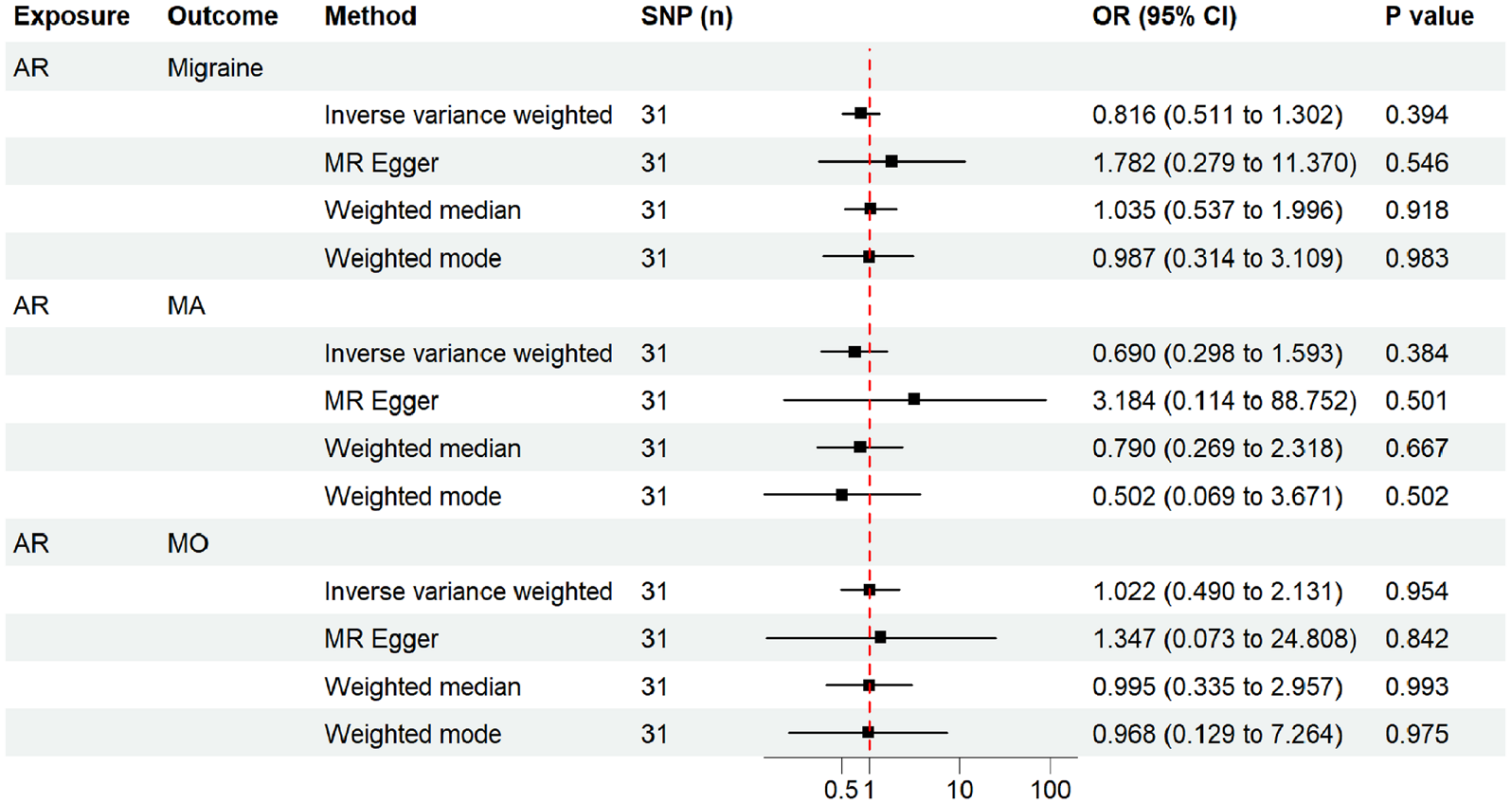
Estimated causal effects of AR on migraine and its subtypes using different MR methods. The scale of x-axis is logarithmic

**Fig. 3:**
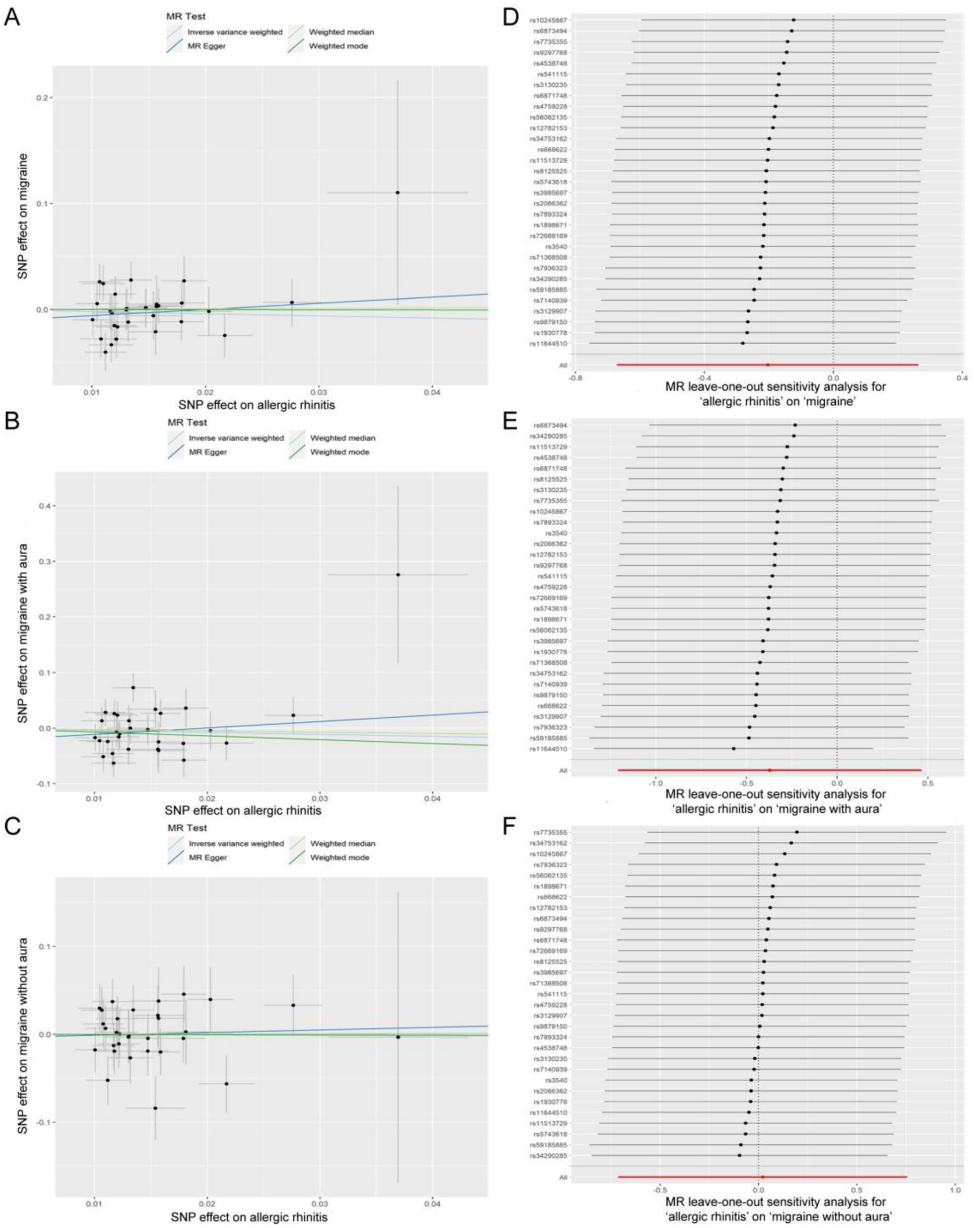
Visualization of the causal effects of AR on migraine and its subtypes. **A** Scatter plot of the causal effect of AR on migraine. **B** Scatter plot of the causal effect of AR on MA. **C** Scatter plot of the causal effect of AR on MO. **D** Leave-one-out sensitivity analysis of the causal effect for AR on migraine. **E** Leave-one-out sensitivity analysis of the causal effect for AR on MA. **F** Leave-one-out sensitivity analysis of the causal effect for AR on MO

The results from the IVW model revealed that genetic predispositions to AR were not associated with an increased risk of migraine (OR, 0.816; 95% CI 0.511–1.302; *P = *0.394) and its subtypes MA (OR, 0.690; 95% CI 0.298–1.593; *P = *0.384) and MO (OR, 1.022; 95% CI 0.490–2.131; *P = *0.954) (Fig. 2). The liability ORs calculated by each method are provided in Additional file 1: Table S5. Analysis with the weighted median, weighted mode, and MR-Egger methods also indicated that AR lacked a genetic causal association with migraine and its subtypes (all *P* > 0.05) (Fig. 2). The results of the MR-PRESSO global test and the MR-Egger intercept test did not reveal the presence of horizontal pleiotropy (all P > 0.05) (Table 2). The funnel plots showing heterogeneity are illustrated in Additional file 2: Fig. S1. The Cochran’s Q-test results revealed no evidence of heterogeneity in the IVs (all *P* > 0.05) (Table 2). The LOO sensitivity analysis demonstrated that the causal association between exposure and outcome was not biased by any single SNP (Fig. 3). These sensitivity analyses confirmed the robustness of the conclusions.

**Table 2:** Sensitivity analyses of the forward MR study

Exposure	Outcome	SNP (*n*)	Heterogeneity tests	Cochran’s Q	*P* value	Horizontal pleiotropy tests	Intercept	*P* value
AR	Migraine	31	IVW	29.28	0.503	MR Egger intercept test	− 0.012	0.400
			MR Egger	28.551	0.489	MR-PRESSO global test	NA	0.395
AR	MA	31	IVW	43.241	0.056	MR Egger intercept test	− 0.023	0.359
			MR Egger	41.986	0.056	MR-PRESSO global test	NA	0.391
AR	MO	31	IVW	26.918	0.628	MR Egger intercept test	− 0.004	0.849
			MR Egger	26.881	0.578	MR-PRESSO global test	NA	0.952

AR, allergic rhinitis; MA, migraine with aura; MO, migraine without aura; SNP, single nucleotide polymorphism; IVW, inverse variance weighted; MR-PRESSO, Mendelian randomization pleiotropy residual sum and outlier; NA, not available

### Causal effects of migraine on AR

**Fig. 4:**
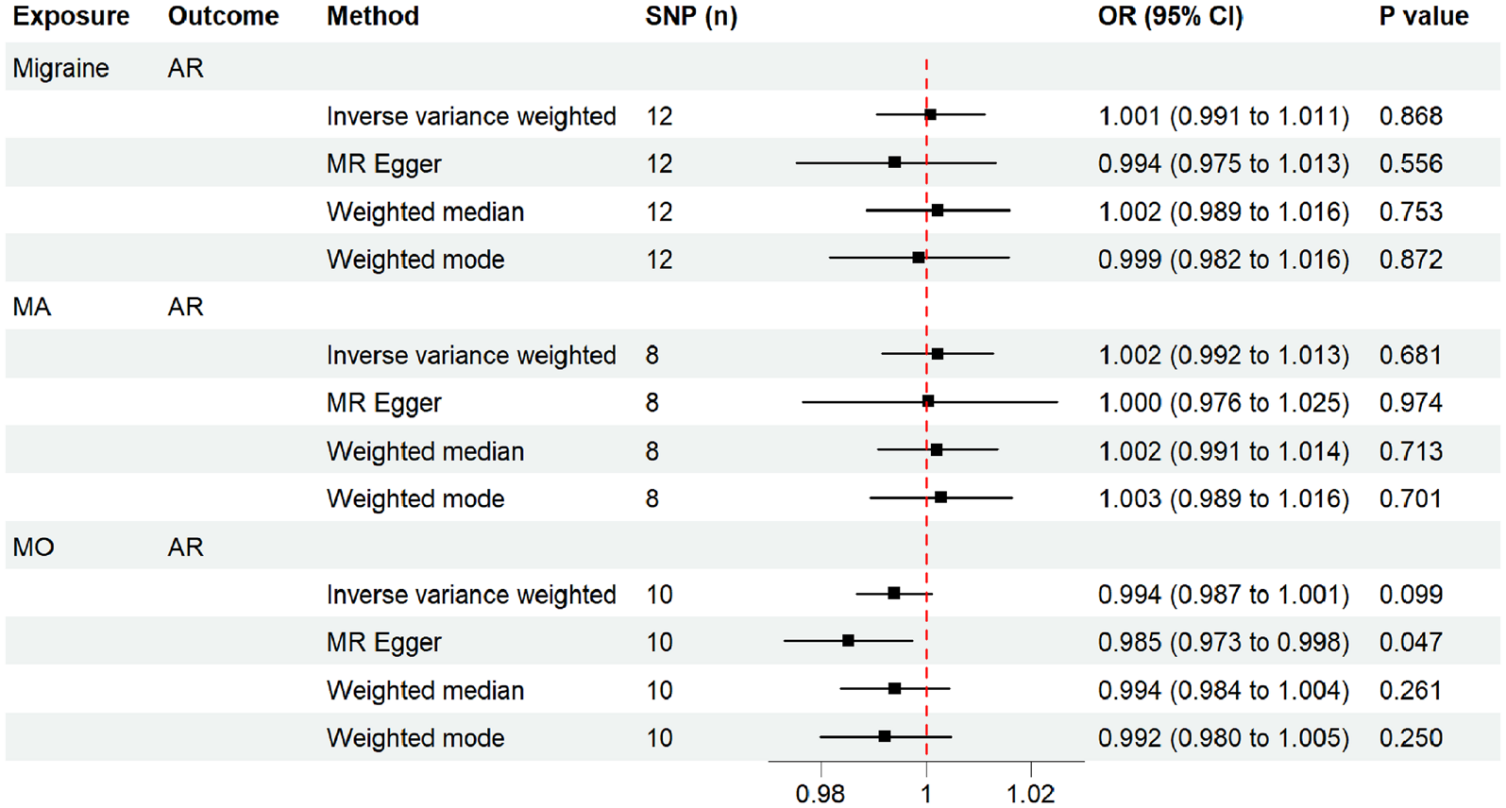
Estimated causal effects of migraine and its subtypes on AR using different MR methods

**Fig. 5:**
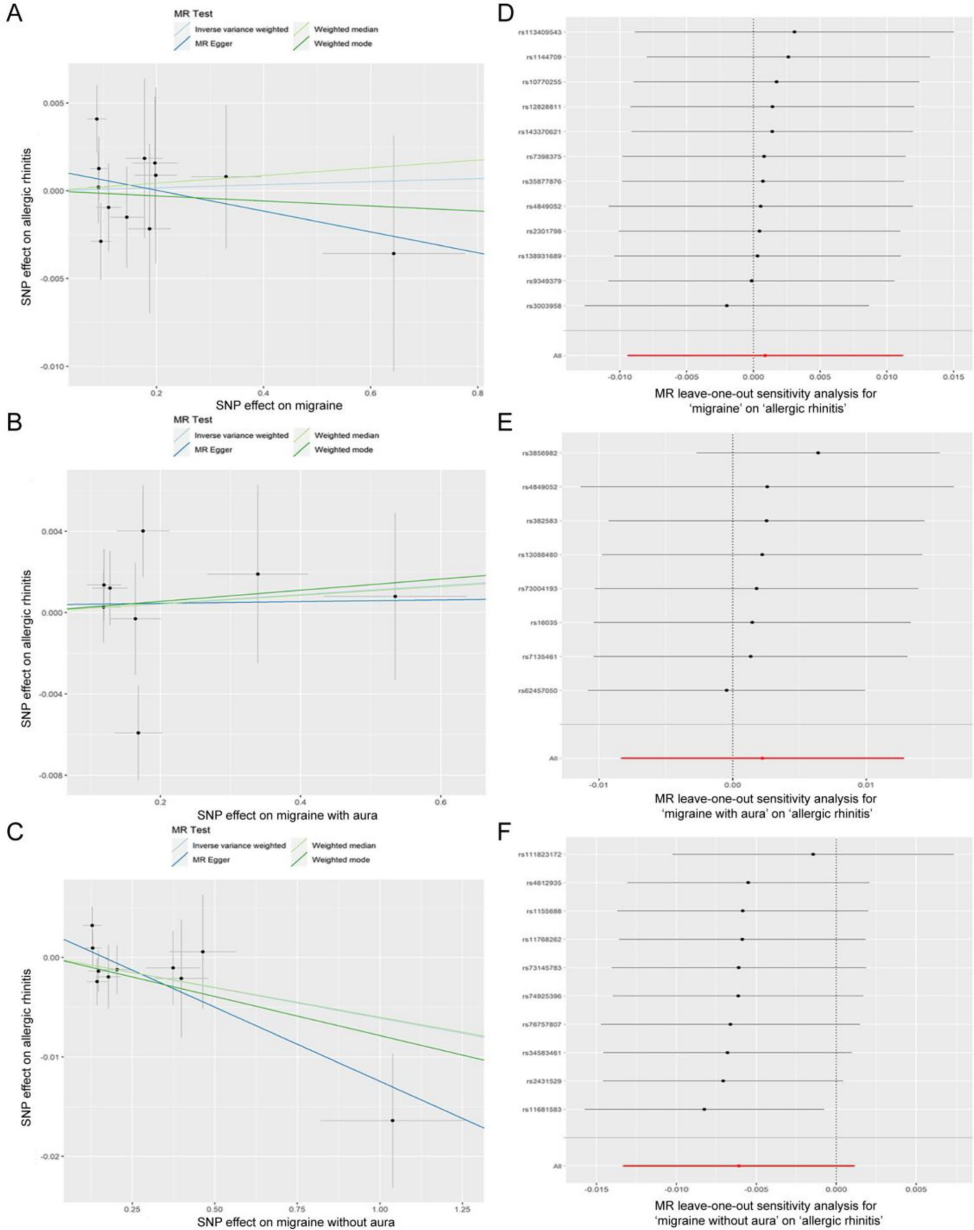
Visualization of the causal effects of migraine and its subtypes on AR. **A** Scatter plot of the causal effect of migraine on AR. **B** Scatter plot of the causal effect of MA on AR. **C** Scatter plot of the causal effect of MO on AR. **D** Leave-one-out sensitivity analysis of the causal effect for migraine on AR. **E** Leave-one-out sensitivity analysis of the causal effect for MA on AR. **F** Leave-one-out sensitivity analysis of the causal effect for MO on AR

To further explore the exact causal relationship between AR and migraine, a reverse MR analysis was performed with migraine, MA, and MO as the exposures and AR as the outcome. Based on the IVW model, migraine (including its subtypes) was not causally correlated with AR (P > 0.05) (Fig. 4). The liability ORs calculated by each method are provided in Additional file 1: Table S5. The results of analysis with the weighted median and weighted mode methods were consistent with those of analysis with the IVW method (all *P* > 0.05) (Fig. 4). The MR-Egger method revealed a possible causal effect of migraine on AR (*P = *0.043). However, the MR-Egger method suffers from an obvious limitation, namely its weak statistical power for detecting a causation [26]. The results of causal analysis with the IVW method are considered the main findings of this study. To further confirm the causal effects of migraine and its subtypes on AR, CAUSE analysis was then performed. The results indicated that the sharing model was better than the causal model, with no significant causal effect of migraine (including its subtypes) on AR (all *P* > 0.05) (Additional file 2: Fig. S2–S4). Furthermore, all sensitivity analyses revealed no significant heterogeneity among the IVs (all *P* > 0.05) and no marked horizontal pleiotropy (all *P* > 0.05) (Table 3). The LOO sensitivity analysis of the causal effect for migraine and MA on AR indicated that no specific SNPs promoted the causal association (Fig. 5). However, the LOO sensitivity analysis assessing the causal effect of MO on AR showed that there was a potentially influential SNP (Fig. 5). Thus, we may need to interpret the conclusion with caution.

**Table 3:** Sensitivity analyses of the reverse MR study

Exposure	Outcome	SNP (*n*)	Heterogeneity tests	Cochran’s Q	*P* value	Horizontal pleiotropy tests	Intercept	*P* value
Migraine	AR	12	IVW	8.118	0.703	MR Egger intercept test	0.001	0.426
			MR Egger	7.430	0.684	MR-PRESSO global test	NA	0.850
MA	AR	8	IVW	10.557	0.159	MR Egger intercept test	0.000	0.874
			MR Egger	10.509	0.105	MR-PRESSO global test	NA	0.693
MO	AR	10	IVW	8.774	0.458	MR Egger intercept test	0.002	0.126
			MR Egger	5.858	0.663	MR-PRESSO global test	NA	0.129

AR, allergic rhinitis; MA, migraine with aura; MO, migraine without aura; SNP, single nucleotide polymorphism; IVW, inverse variance weighted; MR-PRESSO, Mendelian randomization pleiotropy residual sum and outlier; NA, not available

## Discussion

AR and migraine are prominent public health problems worldwide [15]. Many studies have reported the epidemiological overlap between AR and migraine. However, the causal association between AR and migraine is inconclusive [16]. To the best of our knowledge, this is the first bidirectional MR study to investigate the causal association between AR and migraine. The findings of this study indicated no clear causal association between genetic susceptibility to AR and the risk of migraine (including any migraine, MA, and MO). Additionally, migraine (including any migraine, MA, and MO) did not exert a significant causal effect on AR.

Several studies have examined the possible link between AR and migraine. In particular, various observational investigations have revealed a strong epidemiological association between AR and migraine. One case–control study from America involving 76 patients with AR and 57 patients with non-AR revealed that the prevalence of migraine in the AR group was significantly higher than that in the non-AR group [37]. Similar findings were reported by Ozturk et al. [38] who demonstrated that the frequency of migraine in patients with AR was approximately four times higher than that in controls. In 2007, a large cross-sectional questionnaire-based study from Norway revealed that the probability of migraine in patients with hay fever was approximately 1.5 times higher than that in patients with non-hay fever and that this link strengthened with an increase in headache frequency [39]. Additionally, results from the American Migraine Prevalence and Prevention Study (published in 2013) revealed an increased frequency of migraines and disability in patients with AR [12]. Recently, Han et al. [10] performed a nationwide cohort study in a Korean population to investigate the relationship between allergic diseases and migraine risk. Consistent with previous findings, the authors demonstrated that patients with AR had a significantly higher risk of migraine than controls. Additionally, the number of concurrent allergic diseases was positively associated with the risk of migraine. These studies were focused mainly on adults. Some studies have reported a close association between AR and migraine risk in children. Wang et al. [11] performed a nationwide cohort study in a Chinese population involving 461,850 children with AR and 460,718 non-AR controls. The authors demonstrated that the prevalence and subsequent risk of migraine in the AR cohort were significantly higher than those in the non-AR cohort. Additionally, the susceptibility to MO was higher than that to MA in children with AR [11]. Furthermore, several epidemiological investigations have reported that AR may be a potential risk factor for migraine [40]. A retrospective case–control study from Spain reported that the incidence and disease severity of AR in children with migraine were higher than those in children without migraine [41]. A large clinical study conducted by Eross et al. demonstrated that 54% of migraineurs diagnosed according to the criteria of the ICHD had a medical history of AR [42].

The findings of this study are in contrast to those of many observational studies but are consistent with those of some epidemiological studies [13, 14]. This discrepancy may be due to the inherent limitations of observational studies. First, many observational studies in the field are based on case–control and cross-sectional designs, which are ambiguous in terms of chronology, preventing the inference of clear causal relationships. Second, observational studies are susceptible to a variety of confounding factors, even prospective and population-based studies, which may lead to biased results. Third, reverse causation could also lead to observational associations. Finally, the diagnostic criteria used in some studies may reduce the reliability of the findings.

This study did not reveal significant genetic causality between AR and migraine, which must be interpreted with caution. We believe that the association between AR and migraine observed in the clinical setting may be driven by similar pathogenetic mechanisms. Autonomic dysfunction is reported to be one of the potential biological mechanisms contributing to this association [8, 9]. Patients with AR and migraine experience cranial autonomic symptoms, such as rhinorrhea, nasal congestion, lacrimation, and conjunctival injection, which reflect parasympathetic hyperfunction and sympathetic hypofunction [8, 9]. Several basic and clinical studies have reported the important role of parasympathetic hyperactivity in the development of AR and migraine. Furthermore, AR and migraine share the same pathogenic mechanisms, which are based on immune dysfunction and inflammation [10, 11]. The presence of the inflammatory microenvironment in AR may contribute to the aggravation and development of migraine. For example, the levels of many pro-inflammatory mediators involved in AR pathogenesis, such as prostaglandins (PGs), leukotrienes, histamine, nitric oxide (NO), and calcitonin gene-related peptide (CGRP) are significantly upregulated during migraine attack [10, 11]. In addition to mediating allergic inflammation, PGs contribute to pain and inflammation in migraine [43, 44]. CGRP, a neuropeptide released by the trigeminal nerve, plays a crucial role in migraine pathophysiology [4]. Monoclonal antibodies targeting the CGRP system are effective in treating migraine and are considered a breakthrough in migraine pharmacotherapy [45]. CGRP also promotes allergic inflammation by modulating various immune cells, such as group 2 innate lymphoid cells (ILC2s), dendritic cells, and Th2 cells [10]. Histamine released by mast cells is a well-known inflammatory mediator that mediates allergic reactions. Additionally, histamine can increase vascular permeability and NO levels, inducing vasodilation and consequently altering the blood–brain barrier permeability and eliciting localized neurogenic inflammation [11]. These pathophysiological changes are key factors in the development of migraine [4]. Furthermore, the activation and sensitization of the trigeminal system is critical for the development of migraine and AR [46, 47].

One possible explanation for the association between AR and migraine is they share several confounders. Previous studies have demonstrated that several psychiatric disorders, such as depression, anxiety, bipolar disorder, and sleep disturbances are frequently co-prevalent in individuals with migraine and regulate migraine evolution [48, 49]. These psychiatric co-morbidities are strongly associated with increased headache frequency, medication overuse, disability, and poor quality of life in patients with migraine [48, 49]. Furthermore, large-scale GWAS revealed genetic overlap between migraine and psychiatric disorders. Yang et al. [50] reported some same genetic susceptibility loci and significant cross-disorder genetic correlation between migraine and major depressive disorder based on GWAS genotype data. Epidemiological studies have suggested that AR is closely associated with common psychiatric disorders, such as anxiety, depression, bipolar disorder, and attention-deficit/hyperactivity disorder [51, 52]. In both the pediatric and adult populations, the risk of developing psychiatric disorders in patients with AR was significantly higher than that in controls [52, 53]. The severity and duration of AR symptoms were related to poor mental health [52]. Additionally, psychological stress is a common trigger for both AR and migraine. High stress loads induce migraine in susceptible people and increase the frequency of migraine attacks in patients with migraine [54]. Additionally, high stress loads increase the severity of AR symptoms and decrease the efficacy of standard treatments [55].

The major advantage of this study is the application of a robust MR design that minimizes reverse causality and confounding factors associated with traditional observational research. Furthermore, the large-scale GWAS dataset and multiple sensitivity analyses augmented the reliability of the findings. The singular population distribution also effectively diminished the population stratification bias. However, this study has some limitations. First, the GWAS cases in this study were all of European ancestry. Therefore, further studies are needed to determine if the findings of this study can be generalized to other human populations. Second, in-depth stratified analyses, such as sex-stratified and age-stratified analyses, were not performed due to the limitations of the GWAS data. Third, the relatively small number of GWAS samples of migraine subtypes analyzed in this study may lead to decreased statistical power in the reverse MR study.

## Conclusion

This MR study did not provide conclusive evidence to support a direct causal effect between the genetic predisposition to AR and the elevated risk of migraine (including any migraine, MA, and MO). Further studies are needed to elucidate the causal association between AR and migraine risk. We emphasize the benefit of screening patients with AR for migraine and adopting optimal management strategies.

### Supplementary Information

**Additional file 1: Table S1. **Detailed information on the valid IVs associated with AR.** Table S2**. Detailed information on the valid IVs associated with migraine. **Table S3**. Detailed information on the valid IVs associated with MA. **Table S4**. Detailed information on the valid IVs associated with MO. **Table S5**. Liability-scale MR estimates of causal effect between AR and migraine.

**Additional file 2: Figure S1.** Funnel plot of the MR analysis. **A** AR on migraine; **B** AR on MA; **C** AR on MO; **D** migraine on AR; **E** MA on AR; **F** MO on AR. **Figure S2.** Estimated causal effects of migraine on AR using CAUSE analysis. **A** Results of expected log pointwise posterior density (ELPD) and plots of the posterior distributions of the parameters for the sharing model and causal model; **B** Scatter plots of the data showing for each model, the probability that each variant is acting through the shared factor and the contribution of each variant to the ELPD test statistic. **Figure S3.** Estimated causal effects of MA on AR using CAUSE analysis. **A** Results of ELPD and plots of the posterior distributions of the parameters for the sharing model and causal model. **B** Scatter plots of the data showing for each model, the probability that each variant is acting through the shared factor and the contribution of each variant to the ELPD test statistic. **Figure S4.** Estimated causal effects of MO on AR using CAUSE analysis. **A** Results of ELPD and plots of the posterior distributions of the parameters for the sharing model and causal model. **B** Scatter plots of the data showing for each model, the probability that each variant is acting through the shared factor and the contribution of each variant to the ELPD test statistic.

## Data Availability

The datasets used during the current study are available from the corresponding author on reasonable request.
