# Supplementary material for: No causal association between allergic rhinitis and migraine: a Mendelian randomization study
Source: Eur J Med Res. 2024 Jan 27;29:78. doi: 10.1186/s40001-024-01682-1 (PMC10821569; doi:10.1186/s40001-024-01682-1)
Supplement: Supplementary file 1 — Additional file 1: Table S1. Detailed information on the valid IVs associated with AR. Table S2. Detailed information on the valid IVs associated with migraine. Table S3. Detailed information on the valid IVs associated with MA. Table S4. Detailed information on the valid IVs associated with MO. Table S5. Liability-scale MR estimates of causal effect between AR and migraine. [file 40001_2024_1682_MOESM1_ESM.docx]

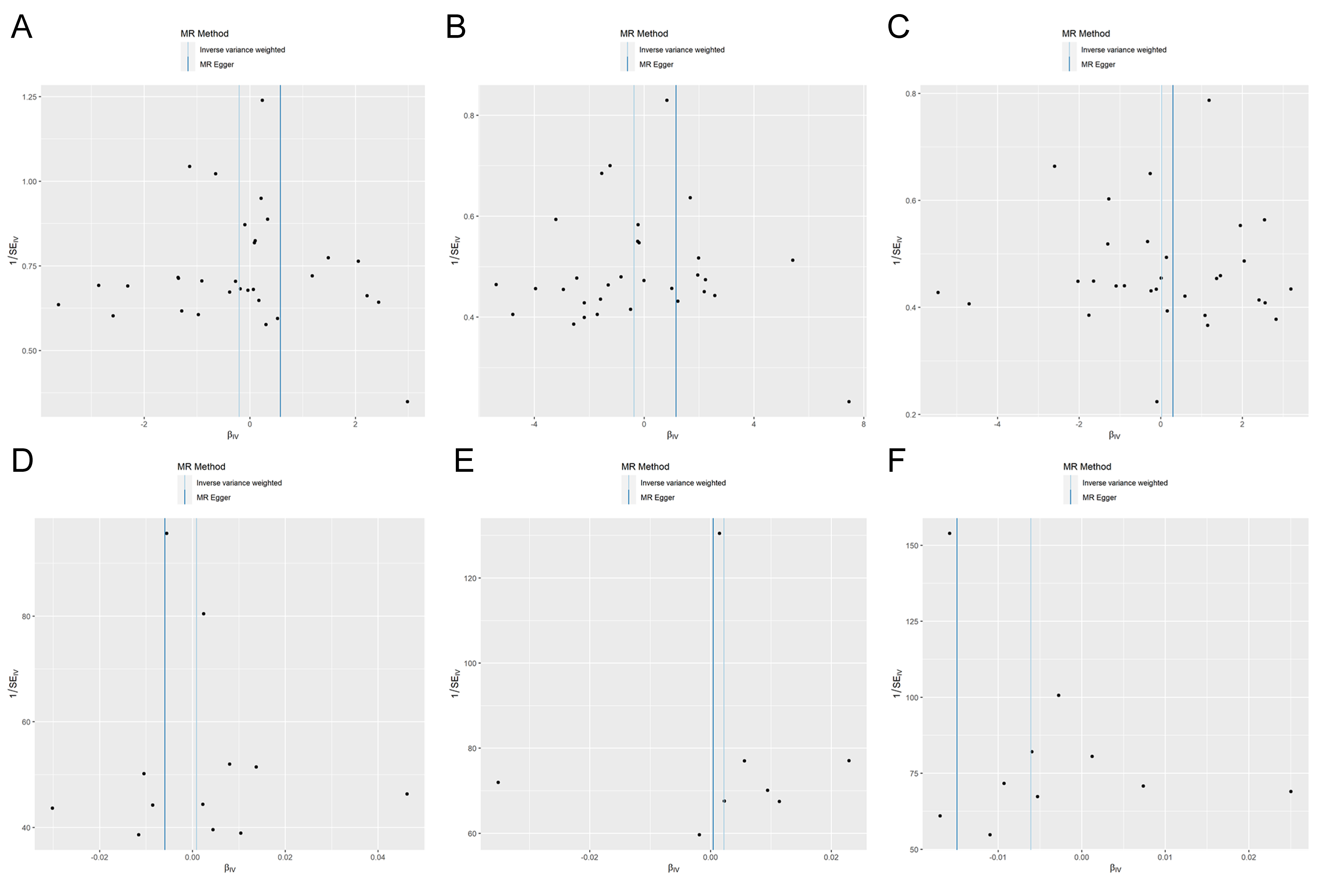


Supplementary Figure 1

Funnel plot of the MR analysis. (A) AR on migraine; (B) AR on MA; (C) AR on MO;

(D) migraine on AR; (E) MA on AR; (F) MO on AR.


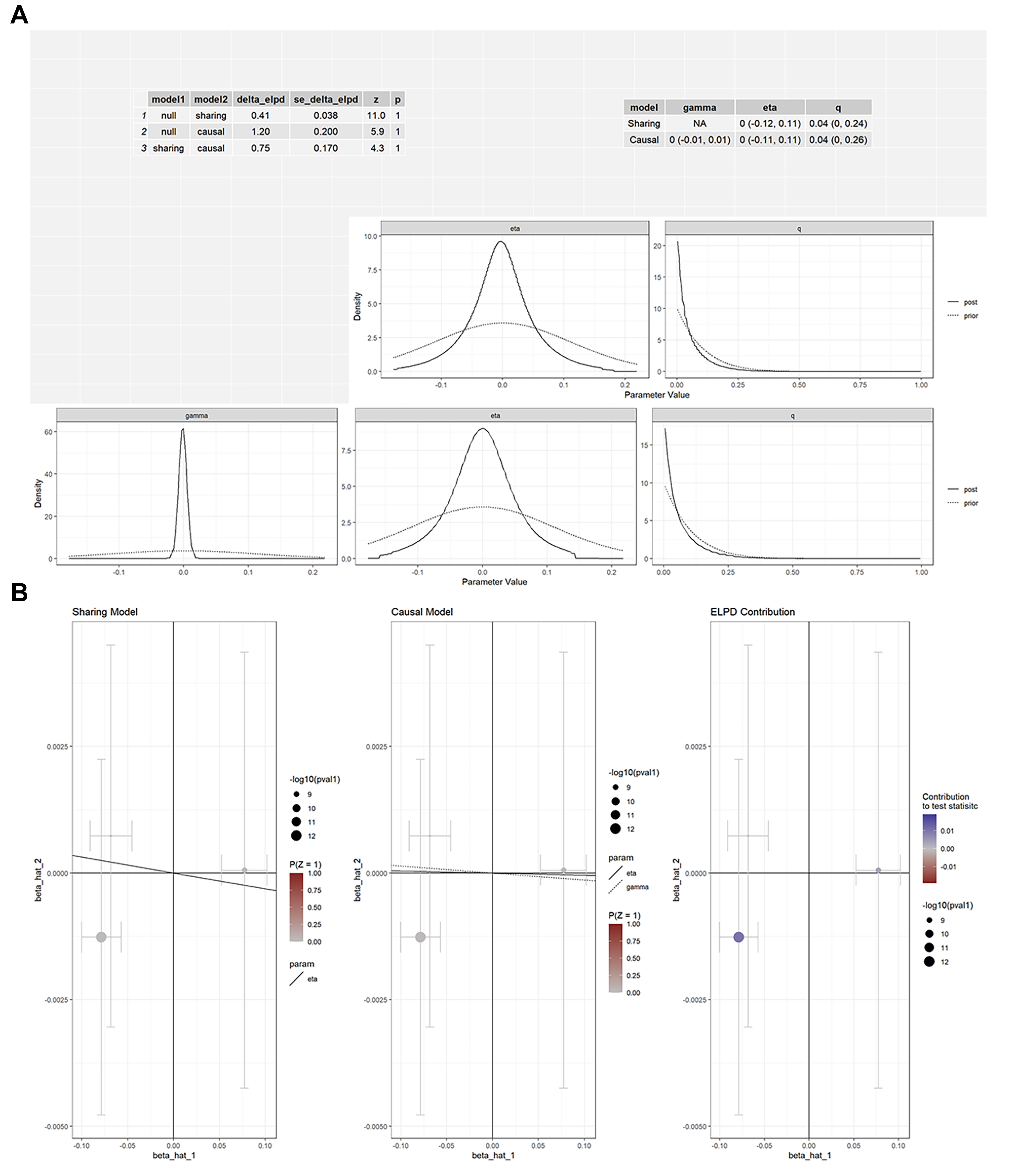


Supplementary Figure 2

Estimated causal effects of migraine on AR using CAUSE analysis. (A) Results of expected log pointwise posterior density (ELPD) and plots of the posterior distributions of the parameters for the sharing model and causal model; (B) Scatter plots of the data showing for each model, the probability that each variant is acting through the shared factor and the contribution of each variant to the ELPD test statistic.


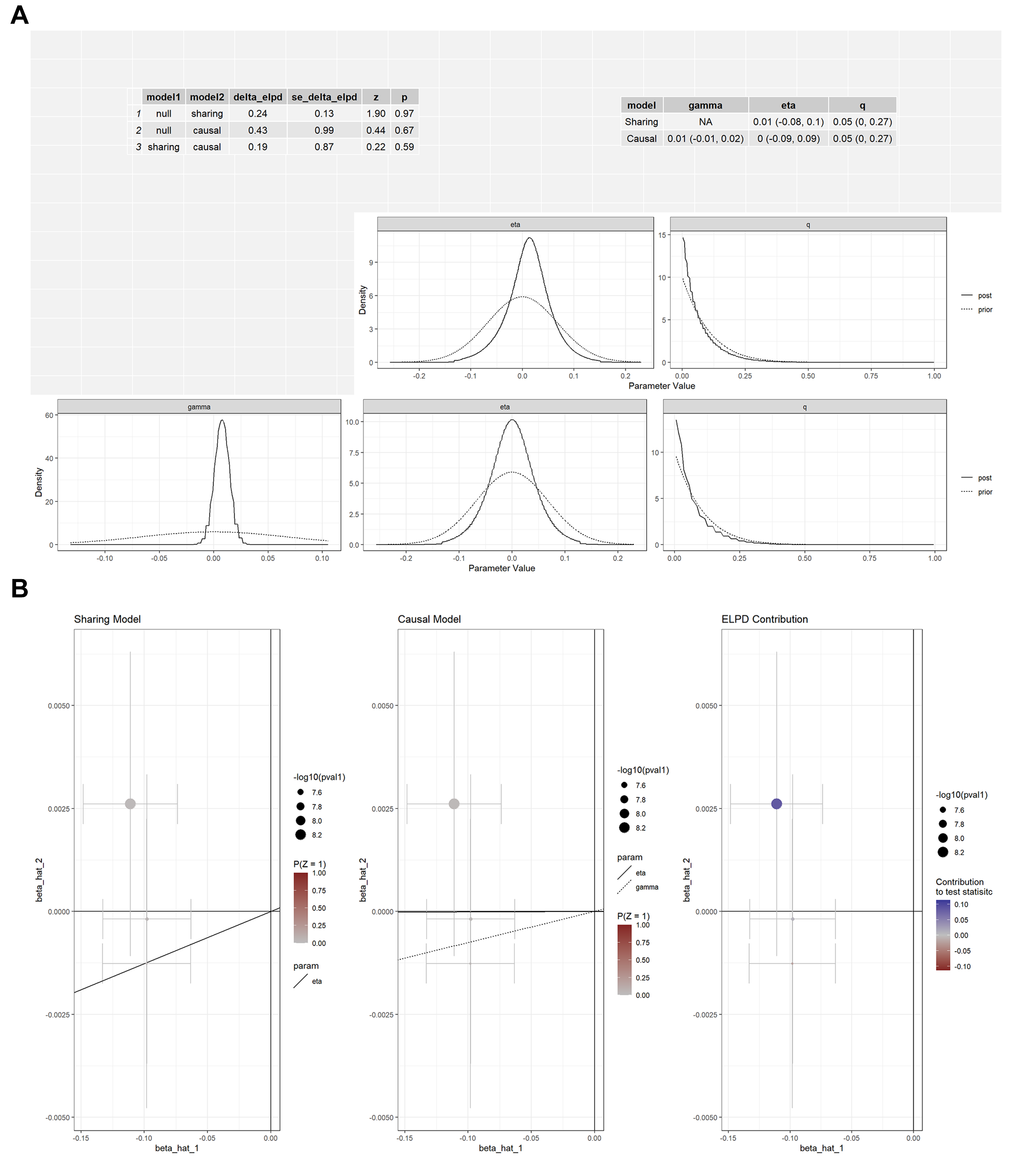


Supplementary Figure 3

Estimated causal effects of MA on AR using CAUSE analysis. (A) Results of ELPD and plots of the posterior distributions of the parameters for the sharing model and causal model; (B) Scatter plots of the data showing for each model, the probability that each variant is acting through the shared factor and the contribution of each variant to the ELPD test statistic.


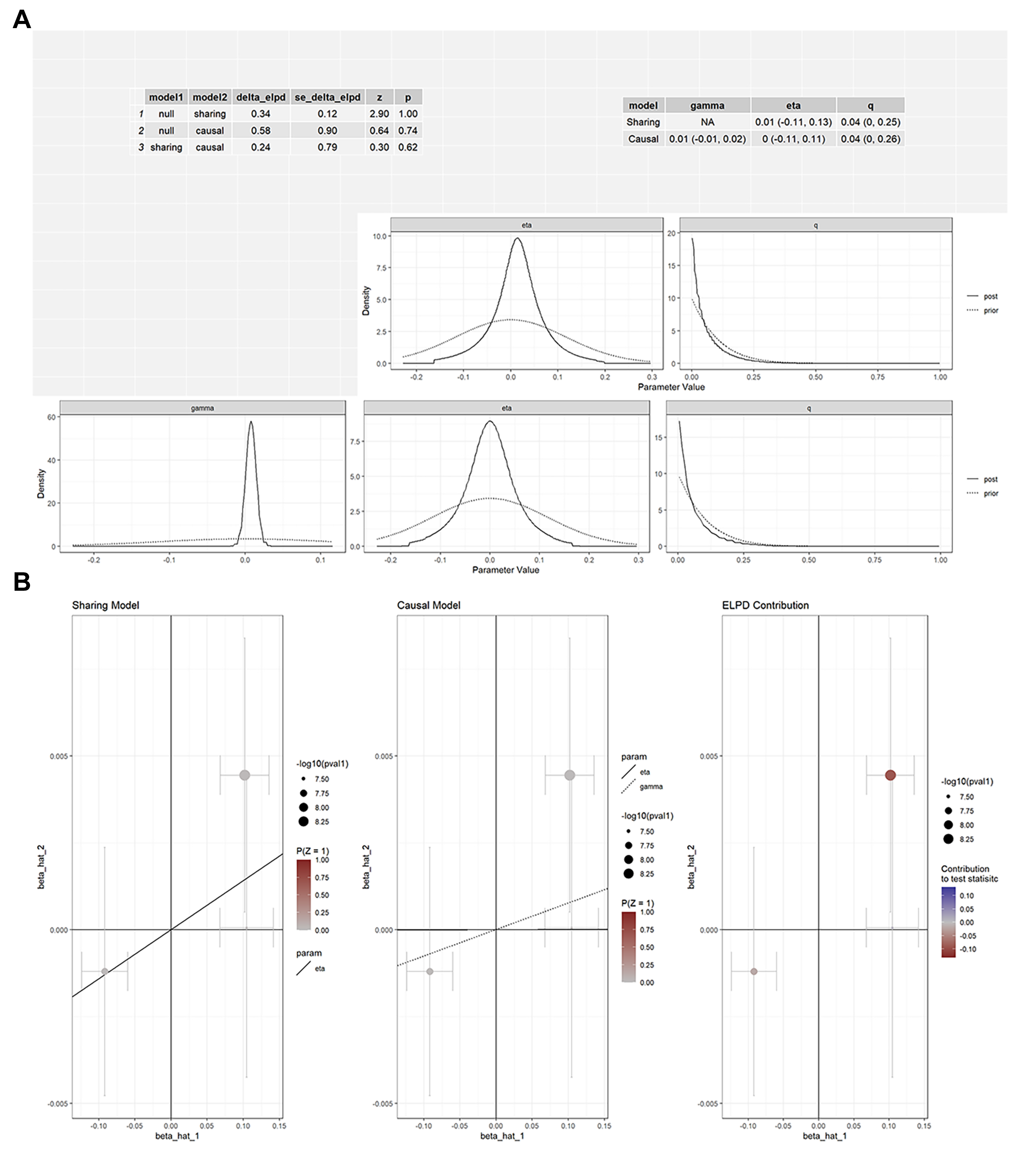


Supplementary Figure 4

Estimated causal effects of MO on AR using CAUSE analysis. (A) Results of ELPD and plots of the posterior distributions of the parameters for the sharing model and causal model; (B) Scatter plots of the data showing for each model, the probability that each variant is acting through the shared factor and the contribution of each variant to the ELPD test statistic.
